# A New Predictive Model for Tattoo Removal: Leveraging Patient and Tattoo Characteristics

**DOI:** 10.1111/jocd.70186

**Published:** 2025-07-19

**Authors:** Candice Menozzi‐Smarrito, Nicolas Pineau

**Affiliations:** ^1^ RIVIERAClinic Switzerland; ^2^ datainsightswiss Lausanne Switzerland

**Keywords:** black tattoo, laser, picosecond, predictive model, tattoo removal

## Abstract

**Introduction:**

The objective of this research was to investigate key parameters impacting the process of tattoo removal and to propose a new predictive model for estimating the number of sessions necessary for the complete removal of black tattoos with a picosecond laser.

**Methods:**

This prospective study involved 116 patients aged 18–62 years who visited our center between January 2020 and June 2024 for the full treatment of black tattoos. Data were collected about patient (age, gender, and phototype), tattoo specifics (age, size, location, ink density, country/region where the tattoo was created, if it was realized by an amateur or a professional tattoo artist, tattoo settings) and the total number of laser treatments. Treatments were performed with a Picosure laser (Cynosure, USA) at 755 nm using fluences of 0.69–6.37 J/cm^2^ a pulsation length of 650 ps. Multi‐way analysis of variance was performed to estimate the effect of each parameter. In order to estimate the number of sessions for complete tattoo removal, a predictive model was then created by the addition of parameter interactions (two‐by‐two only). Additional factors were included or excluded by a stepwise approach (backward and forward). Inclusion and exclusion criteria were based on *p* value thresholds.

**Results and Conclusion:**

ANOVA results revealed that ink density had the most significant impact on laser tattoo removal, followed by tattoo location, age, and design technique (dots, lines, or both). Country/region of origin and whether the tattoo was amateur or professional had a marginal effect, whereas design type (drawing or letters), patient age, skin type, gender, and tattoo size showed no significant influence. Based on these findings, we developed a new efficient predictive tool to estimate the number of picosecond laser sessions required for complete black tattoo removal. The Smarrito–Pineau (SP) model could be readily implemented since variables are easily assessable. It was possible to provide personalized treatment plans and enhance patient satisfaction by offering more accurate treatment timelines.

## 1 Introduction

Tattoos, an ancestral form of artistic expression, have experienced a significant surge in popularity in recent decades. Evolving from simple tribal markings to genuine works of art, tattoos have become democratized and are now a means of personal expression, or even a symbol of belonging to a community [1].

Nevertheless, this burgeoning popularity of tattoos is not without its attendant challenges. Concurrently, we observe a corresponding increase in the demand for tattoo removal procedures [2]. The motivations behind the desire to eliminate a tattoo can be multifaceted, encompassing personal, social, cultural, and professional considerations [3].

Thanks to advances in technology, tattoo removal has become an increasingly safe and effective procedure, allowing those who wish to do so to regain clear skin.

Laser treatment is the gold standard for tattoo removal [4, 5, 6, 7, 8]. Nanosecond Q‐switched alexandrite (755 nm) and neodymium‐doped yttrium aluminum garnet lasers (1064 nm) have been used to remove tattoos with limited side effects. Picosecond (PS) lasers have recently emerged as a promising technology for tattoo removal [9, 10, 11, 12, 13, 14]. Their very short pulse lengths in the PS domain, which are close to the thermal relaxation time of tattoo pigment particles, make it possible to efficiently increase their photomechanical breakup [14]. Thus, PS lasers are gaining significant popularity for tattoo removal due to their effectiveness and faster treatment.

Despite the emergence of effective and reliable technologies to remove tattoos, each tattoo represents a unique challenge due to the numerous factors that can influence their removal. Among these factors, some can be seen as directly related to the tattoo characteristics, to the laser equipment used, or to the patients. The Kirby–Desai (KD) scale was an initial attempt to rationalize key parameters influencing the laser treatment and to provide a useful tool to estimate the required number of sessions to remove a tattoo [15]. The scale was builtrom a retrospective chart review of 100 patients using Q‐switched Nd:Yag or alexandrite lasers. Six parameters were scored for each patient: skin type, location, amount of ink, color, scarring or tissue change, and number of layers. A Pearson's correlation coefficient was performed between the resulting scores and the number of actual tattoo treatments.

However, some parameters of the KD scale are difficult to objectively assess. For instance, scarring and tissue changes were scored from 0 to 5 (minimal to significant). The age of the tattoo was not considered, although several studies have suggested that it could impact the length of the treatment [16, 17, 18]. Moreover, since 2009, laser technology has significantly evolved with the development of the PS laser.

Thus, the objective of the current study was to further assess key parameters impacting laser tattoo treatment and, based on a strong multivariable linear regression analysis, to propose a predictive model estimating the number of sessions required to fully remove a black tattoo using a PS laser.

## 2 Materials and Methods

### 2.1 Design Study

The prospective study involved patients who visited our medical center between January 2020 and June 2024 for treatment of black tattoos. Only patients with Fitzpatrick skin phototypes I–IV who completely removed their tattoos in our medical center were included in this study.

Exclusion criteria when performing laser treatment in our center included hypersensitivity to light exposure or a history of any type of skin cancer. All patients who had ever had any removal treatments on their black tattoo (with laser or any other methods) were excluded from the current panel.

**FIGURE 1:**
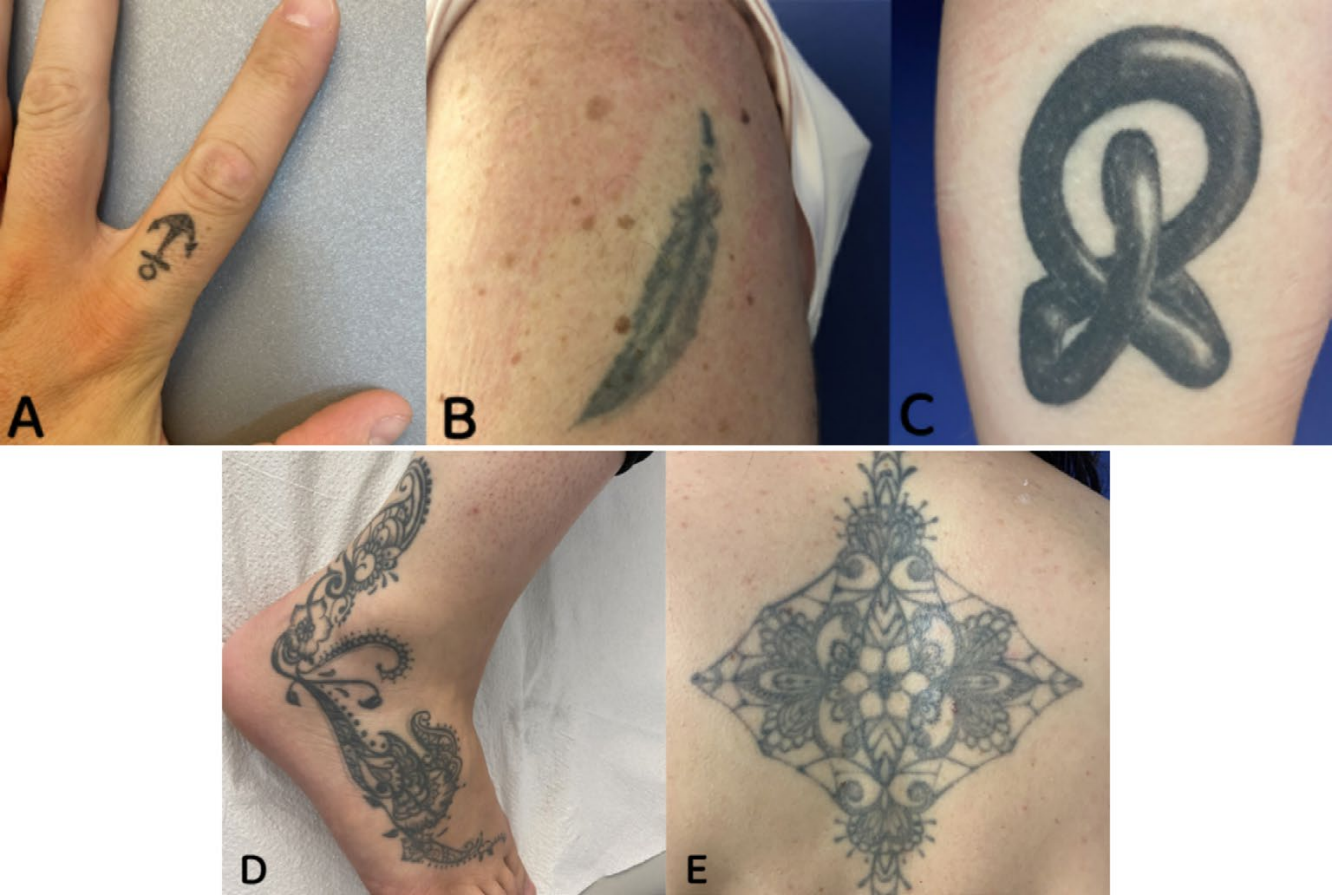
Different tattoo sizes. (A) Very small; (B) small; (C) medium; (D) large; and (E) wide.

**FIGURE 2:**
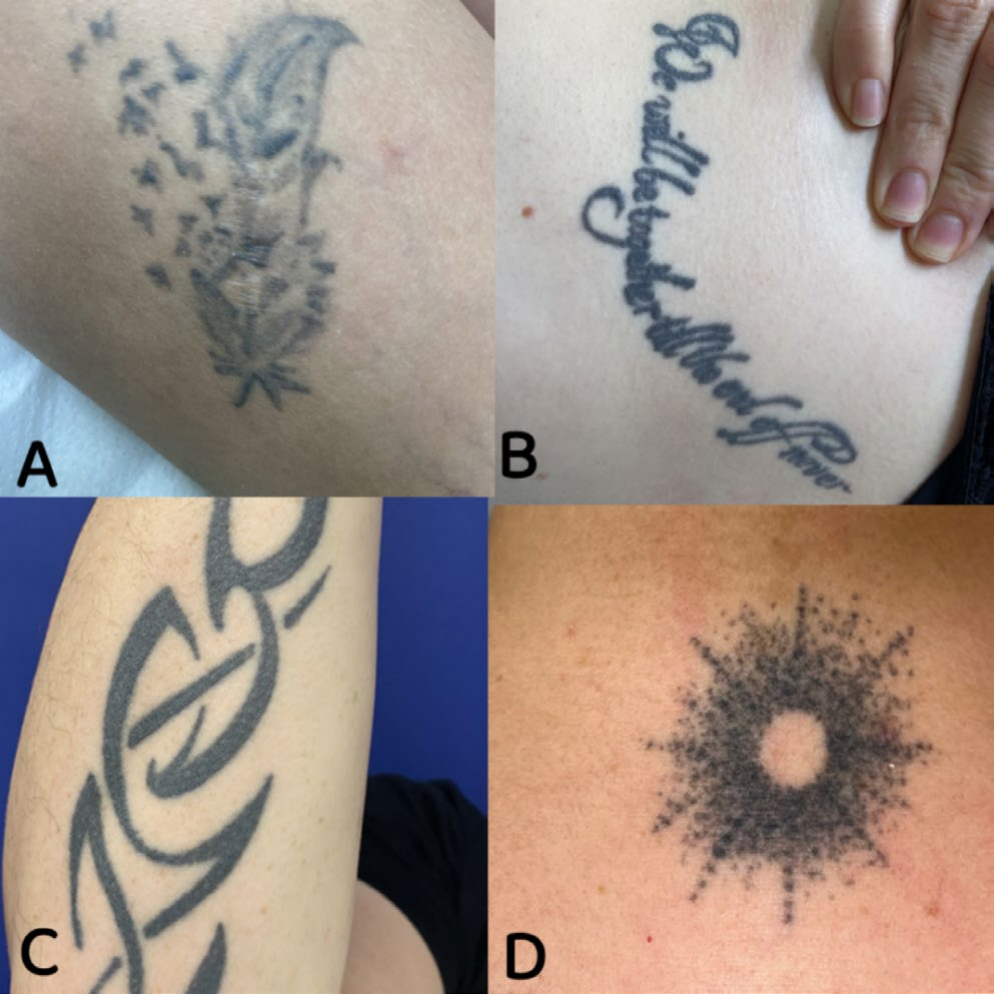
Tattoo characteristics. (A) Low density/mixed/drawing; (B) medium density/lines/letters; (C) medium/mixed/drawing; and (D) dots/drawing.

**FIGURE 3:**
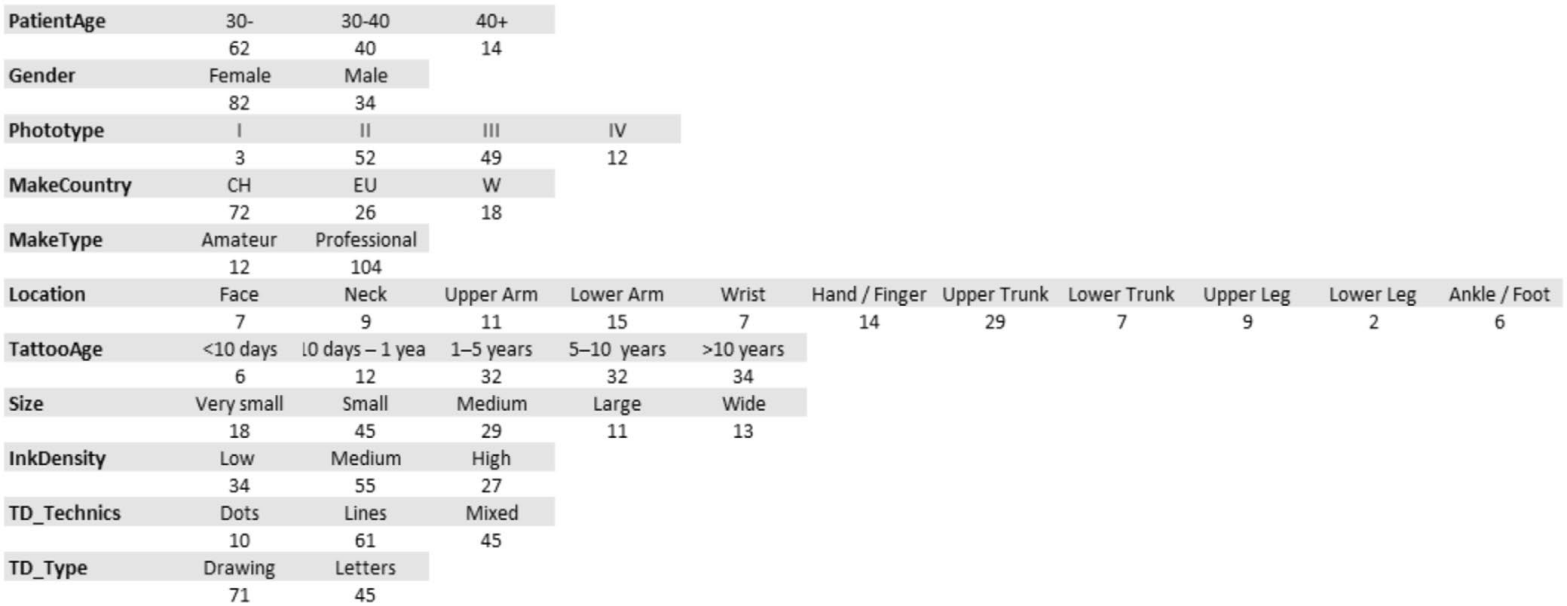
Number of patients per level of each parameter.

Prior to the initial laser treatment, a 30‐min consultation was carried out to ascertain the medical history and explain the procedure. Table 1 summarizes the information collected during the consultation. Figures 1 and 2 show tattoos with different characteristics. Figure 3 describes the population investigated in this study (*n* = 116) for each level of each parameter.

**TABLE 1:** Data collected for each patient.

Name	Description	Categories
PatientAge	Age of the patient at the start of the treatment	Less than 30 years old, between 30 and 40 years old, and more than 40 years old
Gender	The gender of the patient	Female or male
Phototype	The phototype of the patient according to the Fitzpatrick skin type	I, II, III, and IV
MakeCountry	The country or the area where the tattoo was made	CH for Switzerland, EU for Europe and W for the rest of the world
MakeType	Whether the tattoo was an amateur tattoo (including those by nonprofessionals and tattoo apprentices) or a professional tattoo	Amateur or professional
Location	The location of the tattoo	Face, neck, upper arm, lower arm, wrist, hand/finger, upper trunk, lower trunk, upper leg, lower leg, and ankle/foot
TattooAge	The age of the tattoo at the start of the treatment	< 10 days, 10 days to 1 year, 1–5 years, 5–10 years, and > 10 years
Size	The size of the tattoo	Very small (< 2 cm^2^), small (~2–15 cm^2^), medium (~15–64 cm^2^), large (~64–200 cm^2^), and wide (> 200 cm^2^)
InkDensity	The average density of ink of the tattoo	Low, medium, and high
TD_Technics	Main tattoo design technics used to make the tattoo	Dots, lines, and mixed
TD_Type	The tattoo design type	Drawing and letters

### 2.2 Laser Treatments

Informed consent was obtained in writing from all patients prior to the first session. The consent form included authorization to carry out treatment in addition to taking and using photos taken during the sessions. Local anesthesia cream (EMLA, 5% lidocainum/prilocainum) was applied 40 min before the session. Treatments were performed with a Picosure laser (Cynosure, USA) at 755 nm using fluences of 0.69–6.37 J/cm^2^ a pulsation length of 650 ps. Laser parameters were selected based on patient skin type, ink density, and clinical endpoint (whitening). The laser parameters were adapted to each new session, with fluence increasing as the black tattoo was lightened. In the Picosure laser, the higher the fluence, the smaller the diameter of the laser spot (from 5.5 to 2 mm spot diameter). The skin was cooled during the treatment using a Cryo6 system (Zimmer) to make the treatment more comfortable. The interval between treatments was generally 4 weeks at the beginning of the treatment, followed by 8 weeks when a fluence equal to or superior to 5.25 J/cm^2^ was applied, and more than 8 weeks at the end of treatment.

Patients were systematically called 24 h after treatment to obtain data on any side effects (a standard procedure in our medical center). Photos were taken before each session. A follow‐up was carried out 3 months after their last laser session to determine whether the treatment had been completed.

End of treatment was established when there was no more ink on the former tattoo area.

### 2.3 Statistical Analysis

The statistical analysis aimed at understanding the impact of the personal and initial tattoo characteristics (parameters) on the number of sessions required to remove the tattoo (response variable: Nsession). This was conducted in the following two steps:

1. Multi‐way analysis of variance (ANOVA) to estimate the effect of each parameter on the response variable, using type III sum of squares calculation as there were no prior expectations regarding the effect of any parameter.
2. Predictive modeling definition through the addition of parameter interactions (two‐by‐two only). Additional factors were included or excluded by means of a stepwise approach (backward and forward). Inclusion and exclusion criteria were based on *p* value thresholds (with new factor included if *p* value < 0.05, and former factor excluded if *p* value > 0.25). Technical results from the approach were then reviewed by the authors to check for the relevance and explainability of the selected factors. Only the most critical factors were retained in the final model to favor a parsimonious model rather than a more powerful model, which could, however, lead to overfitting.

Statistical calculations were conducted with R software version 4.3.3, using R native functionalities (Base R) as well as the packages leaps and lme4.

## 3 Results

We collected all tattoo characteristics (age, size, location, ink density, country/region where it was made, if it was realized by an amateur or a professional tattoo artist, tattoo technics and type) and completed a full tattoo removal process on 116 patients aged 18–62 years using our PS lasers. The average number of sessions was 6, with a range of 2–20 sessions.

No permanent side effect, such as scars, was observed. In the days following the laser sessions, the formation of bulla, swelling, redness, crusts, and itching were reported. Transient hyperpigmentation and hypopigmentation were observed. Although patients all experienced some side effects, it should be noted that the tattoos located on the ankle took the longest time to heal. In some cases (few tattoos located on the ankle), the laser session had to be postponed because the area was still red after 4 weeks.

### 3.1 Impact of Variables on Tattoo Removal Treatment

The model accounting for all parameter main effects to explain the number of sessions led to a highly significant *p* value (< 0.001), with an *R* ^2^ of 0.63 and an adjusted *R* ^2^ of 0.48. Table 2 shows the results of the ANOVA presented in the material and method section and also shows the parameters sorted by ascending order of *p* value.

**TABLE 2:** ANOVA describing the impact of the parameters on the number of tattoo removal sessions needed to remove a tattoo.

Parameter	df	Sum of squares	Mean square	*F* ratio	*p*
InkDensity	2	328.98	164.49	28.47	< 0.0001
TD_Technics	2	36.46	18.23	3.15	0.0478
TattooAge	4	54.93	13.73	2.38	0.0585
Location	10	108.39	10.84	1.88	0.0602
MakeType	1	10.93	10.93	1.89	0.1727
MakeCountry	2	15.71	7.85	1.36	0.2625
TD_Type	1	1.91	1.91	0.33	0.5665
PatientAge	2	5.94	2.97	0.51	0.6001
Phototype	3	7.60	2.53	0.44	0.7261
Gender	1	0.38	0.38	0.07	0.798
Size	4	5.66	1.41	0.24	0.9121
Error	83	479.63	5.78		

*Note:* Parameters sorted by *p* value in ascending order.

Abbreviations: df, degrees of freedom; *F* ratio, Fisher's statistics.

**FIGURE 4:**
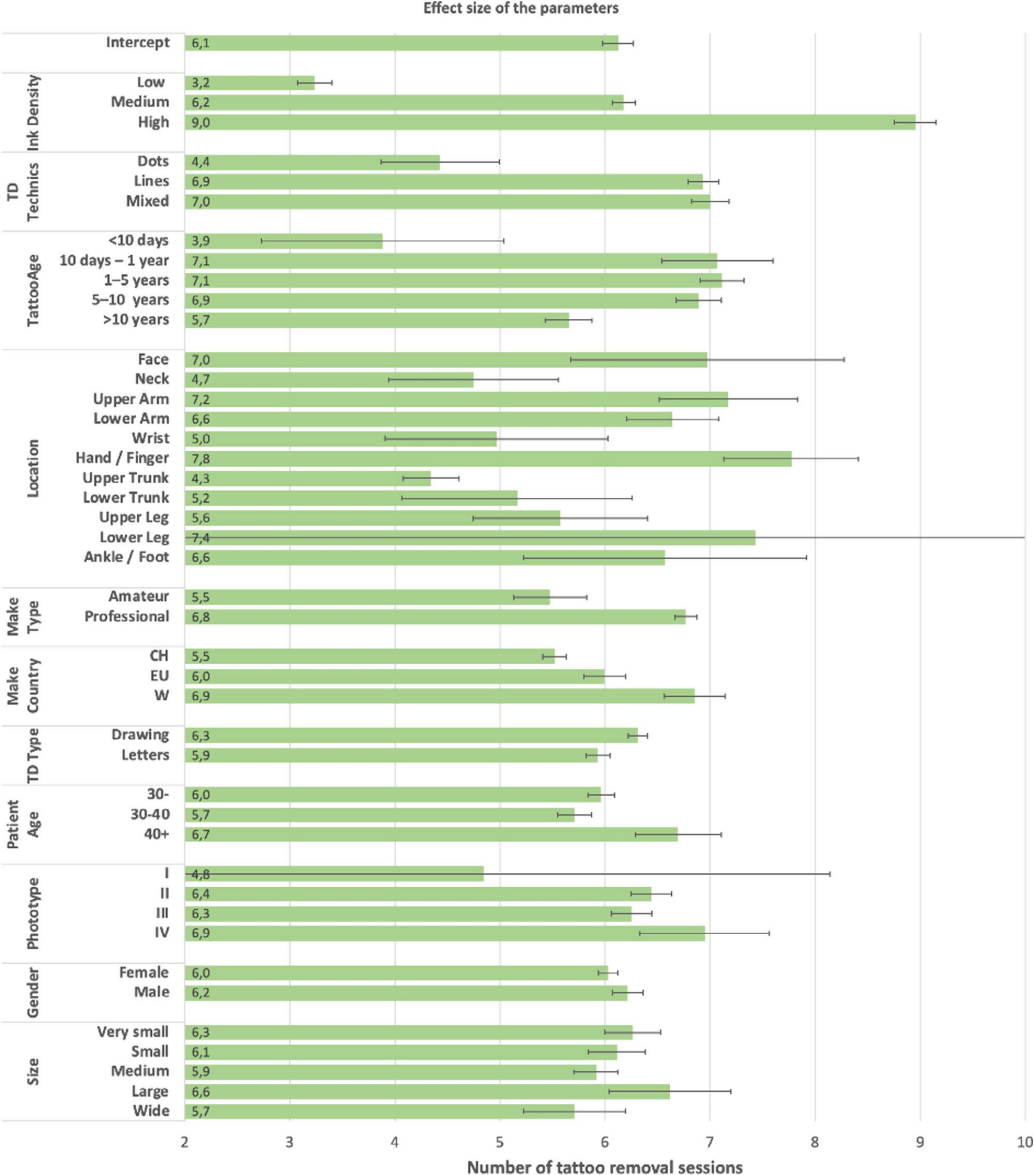
Effect size of each parameter according to the model with all main effects. Error bars = 95% confidence interval of the mean.

Figure 4 shows the adjusted mean values for each level of each parameter (least square means) according to the same model. The most significant effect by far was the ink density (*F* ratio: 28.47, *p* value < 0.0001), indicating that the number of tattoo removal sessions is mostly determined by the density of the ink used to make the tattoo. In other words, the higher the ink density, the higher the number of tattoo removal sessions (from 3.2 sessions on average for low ink density tattoos to 9.0 sessions for high ink density tattoos).

Moreover, the tattoo design (TD) techniques, the age of the tattoo, and the location showed a *p* value that was very close to the usual 0.05 threshold level (resp. 0.0478, 0.0585, and 0.0602), indicating that these parameters also seem to have an impact on the number of sessions. In particular, the dot work technique led to fewer sessions on average (4.4) than the line or mixed ones (resp. 6.9 and 7.0). Regarding the age of the tattoo, those tattoos that were done less than 10 days ago appeared to be the easiest ones to remove, with a significantly lower number of sessions (3.9) than any other age classes. Higher numbers of removal sessions were observed for tattoos older than 10 days, with about 7 sessions on average for tattoos between 10 days and 10 years. After 10 years, the number of sessions was reduced to 5.7. Regarding the tattoo location on the body, one could first observe a much larger size of most of the confidence intervals, indicating much higher variability in the number of tattoo removal sessions for a given location. Nevertheless, the upper trunk part appeared to be the location with the lowest number of removal sessions on average (4.3) and the hand/finger with the highest number (7.8).

At last, even though less important and nonsignificant, the type of tattoo (MakeType, amateur or professional) and the country/area (MakeCountry, Switzerland, Europe, or world) exhibited *p* values below 0.5. The five remaining factors (i.e., the type of design, the age of the patient, the phototype, the gender, and the tattoo size) had very high *p* values (above 0.5) and therefore seem to have no impact on the number of sessions, based on this dataset.

After removing the six parameters having the least impact (size, gender, phototype, PatientAge, TD_Type, and MakeCountry), so that the variability due to them now belonged to the error term, the model was still highly significant (*p* value < 0.001). The *R* ^2^ was only slightly lower (0.61 instead of 0.63) and the adjusted *R* ^2^ even improved (0.53 instead of 0.48). The corresponding ANOVA results, displayed in Table 3, show parameter effects very similar to those from the first analysis, with a slightly more significant *p* value. As this shows, location, tattoo age, and tattoo technics were all significant (*p* value < 0.05), and the *p* values of both the country/region and type of tattoo that were made were both below 0.20. The direction of the effect of each parameter (data not shown) was very similar to the ones displayed in Figure 4 for the first model.

**TABLE 3:** New ANOVA describing the impact of the parameters on the number of tattoo removal sessions needed to remove a tattoo when 6 nonsignificant parameters were excluded.

Parameter	df	Sum of squares	Mean square	*F* ratio	*p*
InkDensity	2	348.60	174.30	32.68	< 0.0001
Location	10	149.56	14.96	2.80	0.0044
TattooAge	4	74.73	18.68	3.50	0.0104
TD_Technics	2	35.94	17.97	3.37	0.0386
MakeCountry	2	19.42	9.71	1.82	0.1676
MakeType	1	9.66	9.66	1.81	0.1816
Error	94	501.37	5.33		

*Note:* Parameters sorted by *p* value in ascending order.

Abbreviations: df, degrees of freedom; *F* ratio, Fisher's statistics.

These two models on the main effect of the parameters have demonstrated a clear impact of the ink density, followed by a significant effect of the tattoo location, tattoo age, and design technics. The country/region of origin of the tattoo and type of tattoo (amateur or professional) had a marginal effect, and finally, the type of design (drawing or letters), the age of the patient, the phototype, the gender, and the tattoo size had no effect.

### 3.2 Predictive Model

Results from the stepwise model selection identified the most impactful interactions to improve the *R* ^2^ statistics in hierarchical order (Table 4). Based on this analysis, the first interaction included was Location*TD_Design, improving the *R* ^2^ from 0.63 to 0.70. Adding the TattooAge*InkDensity interaction on top raises the *R* ^2^ to 0.77, whereas adding Location*Size and, last, TattooAge*Size, respectively, increased the *R* ^2^ to 0.86 and 0.91. However, since the location factor showed relatively high variability of the estimates for each of its levels, its relative interactions (Location*TD_Design and Location*Size) were not kept in order to favor model stability rather than (overestimated) precision. On top, the TattooAge*Size interaction was expected to show at least a logical effect of the tattoo age for each tattoo size, whereas it was not always the case. Typically, estimates were predicting a higher number of removal sessions for tattoos < 10 days compared to older tattoos for some of the levels of tattoo size. A further investigation of the data shows that this result was due to the low number of observations for tattoos < 10 days, with no observations for large and wide tattoos. This interaction was therefore removed as well from the selected model. For completion, and to give an idea of the effect of each interaction on its own, significant or not, the results for all two‐by‐two interactions if added to the model with main effects can be found in Supporting Information.

**TABLE 4:** Hierarchy of best selected models according to the stepwise approach + selected model.

Model #	Model description	df model	df error	*R* ^2^	*R* ^2^ adj.	RMSE	Correl
m1	Main effects	32	83	0.63	0.49	2.404	0.79
m2	m1 + location*TD_Design	42	73	0.70	0.53	2.306	0.84
m3	m2 + TatooAge*InkDensity	50	65	0.77	0.58	2.164	0.87
m4	m3 + Location*Size	70	45	0.86	0.63	2.032	0.93
m5	m4 + TatooAge*Size	82	33	0.91	0.68	1.912	0.95
Selected	m1 + TatooAge*InkDensity	40	75	0.69	0.52	2.332	0.83

Abbreviations: correl, correlation coefficient; df, degrees of freedom; *R* ^2^ and *R* ^2^ adj., *R* squared and adjusted squared; RMSE, root mean square of error.

**FIGURE 5:**
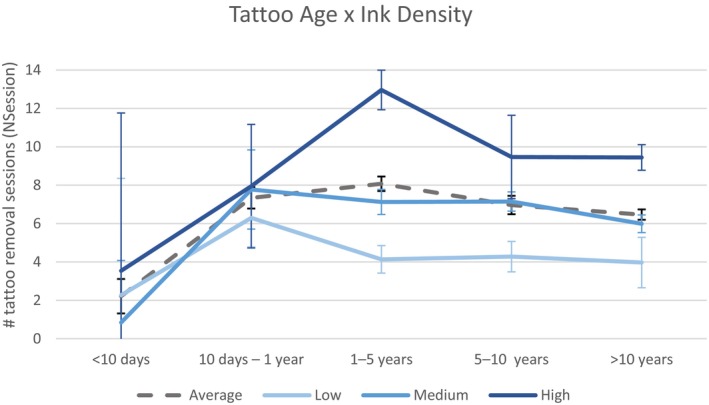
Interaction effect of TattooAge*InkDensity on the number of tattoo removal sessions. Error bars: 95% confidence interval of the average estimates.

As a result, only the TattooAge*InkDensity interaction was kept, as some observations exist for each combination of the main effects, and the results of the interaction were coherent with the main effects, as displayed in Figure 5. The TattooAge*InkDensity interaction shows a similar TattooAge effect shape for each Ink density level, with a minimum number of tattoo removal sessions for tattoos less than 10 days old, a maximum number after this period, and a small decrease after 10 years. However, the interaction reveals that the time at which the number of removal sessions is the highest depends on the ink density, with a shorter age for low density tattoos (maximum number of sessions for 10 days–1 year age category) and an older age for high density tattoos (maximum number of sessions for 1–5 years age category).

The final selected model accounting for main effects (ink density, tattoo location, tattoo age, and design technics) and the TattooAge*InkDensity interaction has an *R* ^2^ value of 0.69 (i.e., corresponding correlation coefficient of 0.83) and an root mean square of error (RMSE) of 2.332.

## 4 Discussion

One of the first questions that patients ask when they want to remove a tattoo is how many sessions they will need. Although the question is simple, the answer is not. Accurately predicting the number of laser sessions to fully remove a tattoo remains challenging since many variables must be considered.

Some parameters that might impact the tattoo removal treatment were previously assessed, and a scale was proposed by Kirby et al. [15]. This scale was based on the amount of ink, the patient phototype, the number of layers of which the tattoo is composed, the occurrence of scarring, the location of the tattoo, and the color of pigments. Their work aimed at rationalizing the key variables to better estimate the number of sessions. However, the technology has greatly evolved, especially with the emergence of PS lasers and other parameters that had not been considered up to now, and this might have a significant impact on laser treatment of tattoos.

We proposed a comprehensive approach, including parameters associated with the patient (age, phototype type, and gender) and to the characteristics of their tattoo (age, size, density, technics and design, location).

Pigments are found in the dermis as free particles and in membrane‐bound granules within macrophages, fibroblasts, and mast cells [19]. A photoacoustic effect induced by laser light causes the rupture of granules. Tattoo pigments are thus broken into smaller particles. Phagocytosis occurs through the lymphatic system, and ink particles small enough are eliminated, causing progressive fading. The maximal diameter of particles that can be absorbed by the lymphatic system is around 0.4 μm [20]. The lymphatic system therefore plays an important role in the tattoo removal process. Since the effectiveness of the lymphatic system might decrease with age, one would expect that the older the patient, the more sessions he/she would need to remove a tattoo. Our results show that, although patients who were more than 40 years old at the start of the treatment tend to need more sessions on average than younger patients (6.7 vs. 6.0, Figure 4), the effect of age was not statistically significant. Also, no significant impact of patient gender was observed.

Kirby et al. included the phototype in their scale to estimate the number of laser sessions with a score from 1 to 6, the highest score corresponding to the dark skin phototype VI. In our study, the skin type did not have a major impact on laser tattoo treatment. Among patients with a phototype IV, there was a slight tendency toward requiring more sessions than skin types I, II, and III, but it was not statistically significant. We did not include patients with phototypes V and VI, since the PS 755 nm laser is not suitable for dark skins. However, the phototype of the patient would remain a key variable to define suitable laser parameters and the risk of adverse effects, with a high phototype being more prone to hyperpigmentation or hypopigmentation.

ANOVA has shown that location, tattoo age, and TD techniques were all significant parameters influencing the number of laser sessions. Although the impact of location was previously investigated, tattoo age and techniques had not been assessed yet. Kirby et al. [15] associated the location of the tattoo with the lymphatic network that is involved during laser tattoo removal. The more the lymphatic system is deployed on the tattoo area, the more effectively it can eliminate pigment particles after the session. Thus, Kirby et al. classified the area assigned in order of ease as follows: head/neck and upper trunk, followed by lower trunk, proximal, and distal area. Our research reported a significant effect of the tattoo location on its removal, which globally is in line with previous studies. The neck, the upper and lower trunk, as well as the upper legs were more favorable than the ankles, feet, hands, fingers, arms, and the lower legs. Interestingly, tattoos located on the face were found to be harder to remove than those on the neck, and those on the wrist required a similar number of laser sessions as those located on the trunk. Our outcomes thus confirm the importance of the lymphatic supply in the process of tattoo removal, although other physiological factors might also affect the number of laser sessions needed to remove a tattoo.

When tattooing, pigments were injected below the dermal‐epidermal junction, at the level of the papillary dermis. Tattoo wound healing includes immediate pigment shedding through the epidermis, long‐term ink retention by dermal macrophages, and gradual transport via lymphatics to deeper tissues. Kirby et al. did not include the age of the tattoo as a variable to estimate the number of tattoo removal sessions. The impact of tattoo age has not yet been clearly defined. Based on the fact that the ink particles migrate deeper into the reticular layer of the dermis over time, the removal of old tattoos was considered to be more difficult and requiring more laser sessions [17]. Conversely, Ferguson et al. [16] suggested that older tattoos may need fewer sessions as some ink particles naturally fade. In our study, we observed a significant impact of the age of the tattoo: Fewer laser sessions were needed when tattoos were very recent (less than 10 days) and older than 5 years. However, treatment feasibility on very recent tattoos should be assessed on a case‐by‐case basis by assessing the condition of the skin. Moreover, tattoos that were more than 5 years old faded over time due to the permanent action of the lymphatic systems. This means they would require fewer laser sessions, which is in accordance with Ferguson et al. [16].

**FIGURE 6:**
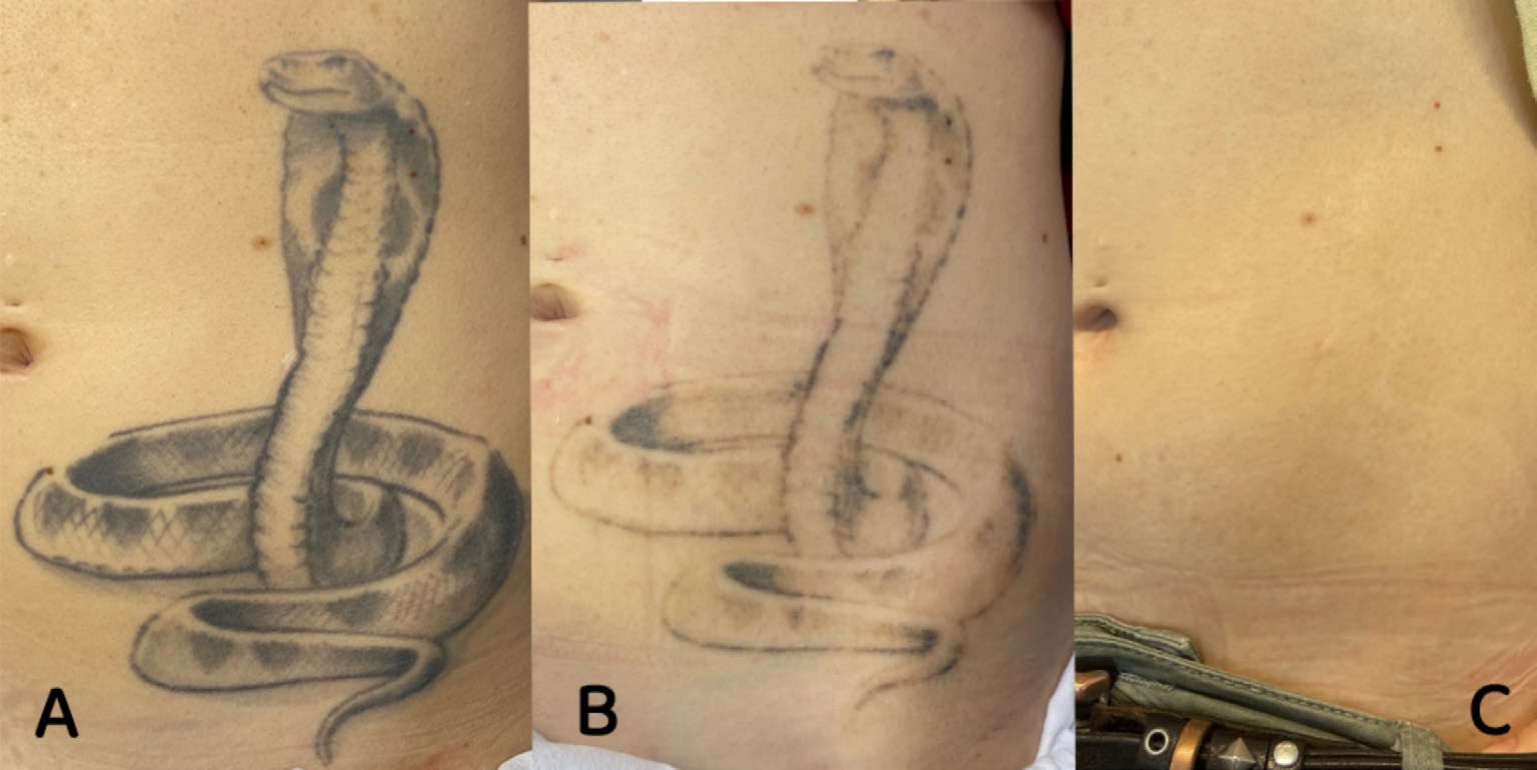
(A) Before treatment; (B) after 1 laser session; and (C) after 5 laser sessions.

Based on our statistical analyses, the size of the tattoo had no significant impact, whereas the ink density was the major parameter influencing the laser treatment: The denser the tattoo, regardless of its size, the higher the number of sessions. The density is related to the amount of pigment and at larger extend the depth. For instance, considering the tattoo depicted in Figure 6, shadings that were of low ink density deposited shallowly could be removed more easily than lines. Kirby et al. included in their scale simple tattoos (1 layer) and cover tattoos (several layers generally created by different tattoo artists). We did not include cover tattoos in our current study, although they would be categorized logically into the “high ink density” requiring a higher number of laser sessions that is in accordance with Kirby et al.

In our model, we chose to separate the design (drawing and letters) and the techniques that the tattoo artists had employed (dots, line, and mixed). Only techniques significantly influenced tattoo removal: Dotwork tattoos (as well as those with shadings) were easier to remove than those with lines or mixed, which can be linked to the density and the depth of tattoo particles.

Finally, the experience of the tattoo artist (amateur/apprentice vs. professional) did not significantly impact the number of laser sessions needed to remove a tattoo. However, what we observed during our practice was that professional tattoo artists would inject ink uniformly in the dermis, whereas the amateurs might inject ink either too deeply or too superficially due to their lack of experience. Thus, tattoos made by unexperienced artists might result in uncertainties in the treatment.

Regarding the parameter country/area, we had hypothesized that ink regulations that vary significantly across countries and geographic regions might influence the effectiveness of tattoo removal procedures. However, to rigorously evaluate this hypothesis, we would require a substantially larger dataset encompassing global tattoo ink usage. Furthermore, it is important to acknowledge that the use of inks within a specific country does not necessarily guarantee their compliance with local regulations. In some instances, guest tattoo artists from other countries may bring their own materials, making it extremely difficult to ascertain the origin and the quality of the inks used.

### 4.1 Predictive Model Estimating the Number of Laser Sessions

Our large panel of data allowed us to build a predictive model with a strong correlation coefficient considering the ink density, tattoo location and age, and tattoo technic. In order to further assess the effectiveness of our model, 12 patients/tattoos were randomly selected, and the predictions of the required number of laser sessions were compared between the KD and Smarrito–Pineau (SP) models. To ensure a fair comparison, the 12 patients/tattoos selected for validation were not used to build the SP model. As shown in Table 5, the SP model provided a more accurate estimation compared to the KD model with a lower RMSE, demonstrating the robustness and efficiency of our predictive model. To explain further, the RMSE of 3.7 observed for the KD model means the prediction is deviating on average by ±3.7 sessions from the true value, whereas the SP model is deviating only by ±2.2 sessions, which corresponds to an improvement of 41% in the model prediction.

**TABLE 5:** Comparison between Kirby–Desai (KD) and Smarrito–Pineau (SP) models to predict the number of laser session to remove a black tattoo.

Tattoo	KD theoretical number of sessions	SP theoretical number of sessions (rounded)	Experimental number of sessions (target)	Delta^2^ KD	Delta^2^ SP
1	10	11	11	1	0
2	12	10	11	1	1
3	7	5	6	1	1
4	12	8	5	49	9
5	5	7	6	1	1
6	7	4	6	1	4
7	12	6	4	64	4
8	10	6	5	25	1
9	12	7	8	16	1
10	7	4	5	4	1
11	10	10	9	1	1
12	8	7	8	0	1
			RMSE	3,7	2.2

Abbreviation: RMSE, root mean square of error.

## 5 Conclusion

This paper proposes a comprehensive study to identify the key factors affecting the number of laser sessions required for tattoo removal. Statistical analyses revealed that ink density, tattoo location, age, and the artist's technique significantly influenced the removal process.

Based on these findings, we developed an effective predictive tool to estimate the number of PS laser sessions needed to completely remove a black tattoo. Although it is impossible to determine the exact number, our SP model might offer a more accurate and reliable prediction than the traditional KD scale. This SP tool could empower patients to make informed choices before embarking on tattoo removal. It is crucial to include information on potential complications, as they occur in approximately 6% of all tattoo‐related complications [21]. This transparency allows patients to make well‐informed decisions.

It is worth mentioning the Smarrito–Pineau predictive model has been developed on one initial dataset with 116 data points for now. Even though cross validation was used to ensure more robust predictions, additional data could be collected in the future to improve predictions. The same scope (black tattoos) might be considered but adding data from more practitioners and medical centers, including cover tattoos, and patients of phototypes V and VI. Enlarging the scope of the tool by targeting different types of PS lasers and even colored tattoos might be another axis of development.

## Disclosure

The authors assure that for this manuscript, the following is fulfilled: This material is the authors' own original work, which has not been previously published elsewhere. The paper reflects the authors' own research and analysis in a truthful and complete manner. All authors have been personally and actively involved in substantial work leading to the paper and will take public responsibility for its content.

## Consent

The patient's consent has been obtained (patients' information and images).

## Conflicts of Interest

The authors declare no conflicts of interest.

## Data Availability

The data that support the findings of this study are available from the corresponding author upon reasonable request.
